# Global trends in research on the effects of climate change on *Aedes aegypti*: international collaboration has increased, but some critical countries lag behind

**DOI:** 10.1186/s13071-022-05473-7

**Published:** 2022-09-29

**Authors:** Ana Cláudia Piovezan-Borges, Francisco Valente-Neto, Gustavo Lima Urbieta, Susan G. W. Laurence, Fabio de Oliveira Roque

**Affiliations:** 1 Instituto de Biociências (INBIO), Universidade Federal de Mato Grosso do Sul (UFMS), Campo Grande, Mato Grosso do Sul Brazil; 2 Laboratório de Mamíferos, Departamento de Sistemática e Ecologia, Programa de Pós-Graduação em Ciências Biológicas (Zoologia), Universidade Federal da Paraíba (UFPB), João Pessoa, Paraíba Brasil; 3 Centre for Tropical Environmental and Sustainability Science (TESS), College of Science and Engineering, James Cook University, Cairns, Australia

**Keywords:** Temperature effects, Primary dengue virus vector, International collaboration, Bibliometric analysis

## Abstract

**Background:**

Mosquito-borne diseases (e.g., transmitted by *Aedes aegypti*) affect almost 700 million people each year and result in the deaths of more than 1 million people annually.

**Methods:**

We examined research undertaken during the period 1951–2020 on the effects of temperature and climate change on *Ae. aegypti*, and also considered research location and between-country collaborations.

**Results:**

The frequency of publications on the effects of climate change on *Ae. aegypti* increased over the period examined, and this topic received more attention than the effects of temperature alone on this species. The USA, UK, Australia, Brazil, and Argentina were the dominant research hubs, while other countries fell behind with respect to number of scientific publications and/or collaborations. The occurrence of *Ae. aegypti* and number of related dengue cases in the latter are very high, and climate change scenarios predict changes in the range expansion and/or occurrence of this species in these countries.

**Conclusions:**

We conclude that some of the countries at risk of expanding *Ae. aegypti* populations have poor research networks that need to be strengthened. A number of mechanisms can be considered for the improvement of international collaboration, representativity and diversity, such as research networks, internationalization programs, and programs that enhance representativity. These types of collaboration are considered important to expand the relevant knowledge of these countries and for the development of management strategies in response to climate change scenarios.

**Graphical Abstract:**

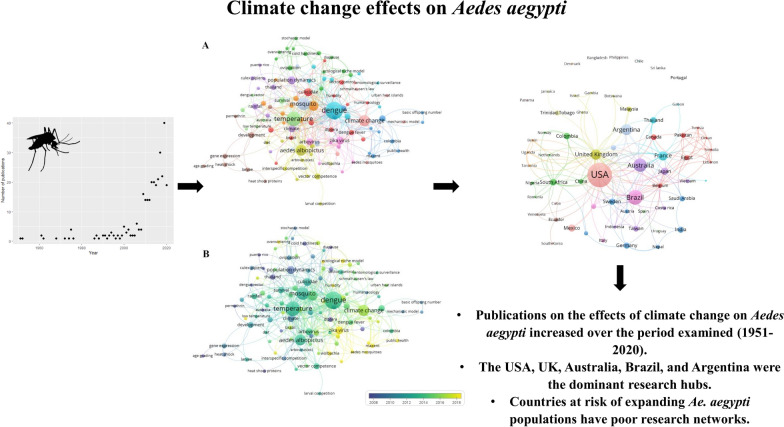

**Supplementary Information:**

The online version contains supplementary material available at 10.1186/s13071-022-05473-7.

## Background

*Aedes aegypti* is one of the main species responsible for the transmission of mosquito-borne pathogens worldwide. These pathogens include the viruses that cause Zika virus disease, chikungunya fever and dengue fever [1, 2], which place a heavy burden on human communities. Changes in climate, such as temperature, induce alterations in insect development [3–5], reproduction [5, 6], and behavior [7, 8]. A number of studies have investigated the potential effects of temperature or climate change on life history traits of *Ae. aegypti* or on epidemiological factors related to this mosquito [9–13]. An increase in temperature accelerated larval development [5] and adult emergence [9] and decreased the period of extrinsic incubation (the period of pathogen development inside the vector until it is capable of being transmitted) [14] and increased the global distribution of *Ae. aegypti* in climate change scenarios [15, 16]. These findings are key to understanding how *Ae. aegypti* responds to climate change and the consequences of this for human health.

Many developing countries have ideal climatic conditions for *Ae. aegypti* development and struggle with diseases transmitted by this species. This is due to a number of factors, including a lack of basic health services, precarious public health systems, population growth, and intensive and uncontrolled urbanization [17–19]. Climate change can further exacerbate these problems, with an increase in the frequency and occurrence of extreme weather events, such as prolonged drought or flooding, impacting communities in affected areas [20], and a rise in temperature increasing the number of diseases, such as vector-borne diseases, the incidence of epidemics of water-borne diseases, and the occurrence of cardiovascular disease [18]. Consequently, poorer countries and people are likely to be most affected by the effects of climate change [21]. Understanding trends in research on the effects of climate change on *Ae. aegypti* is essential to improving the role of science in helping nations to overcome the threats posed by this species.

International scientific collaborations are highly beneficial for both developed and developing countries. They can improve research quality through an increase in the critical mass of researchers [22], and may also lead to an enhancement in writing skills [23], which may help achieve publication in high impact journals and generate greater visibility of research results [24]. Furthermore, the establishment of good relationships among countries can help to generate partnerships for future research and opportunities for students, and aid problem solving [22]. Historically, the USA and Europe have had the largest collaboration networks [25, 26]; however, these may have resulted from “helicopter research,” whereby wealthier countries collect data from low-income countries but do not involve local scientists in the research [27]. However, in recent years, many developing countries have increased their internationalization, with connections between national and international researchers promoted by the scientific policies of governmental and private funding agencies [24]. Yet, despite some improvement in representativity (e.g., diversity, equity and inclusion), it is important to note that transformative actions are urgently required for scientific communities around the world to enhance diversity, equity and inclusion at author, leadership, and societal levels [28].

Understanding the global pattern of research on *Ae. aegypti* in the context of climate change is essential because, by anticipating the impacts of this species, it can lead to an improvement in global responses. A lack of participation of certain countries in this research may lead to misalignment between the solutions proposed through research and the reality of what different countries actually experience. This is true not only for developing countries located in the regions with the highest occurrences of *Ae. aegypti* [29] but also for developed countries where the species is not currently present but is predicted to occur through range expansion according to climate change scenarios [15]. For this overview, we explored the literature using bibliometric analysis to summarize global patterns regarding the effects of temperature and/or climate change on *Ae. aegypti.* We examined the number of publications, research trends and collaborator networks. Furthermore, as the number of publications from a country may vary according to factors related to human health (e.g., number of dengue cases) and socioeconomic factors, we assessed the relationships between the number of publications per country and dengue cases and socioeconomic indices [Gini coefficient, gross domestic product (GDP) per capita and human development index (HDI)].

## Methods

### Literature search, inclusion and exclusion criteria

We searched the Institute of Scientific Information Web of Science database and retrieved all articles from the first insertion until October 2020. Our search terms were (“climate change” OR “temperature” OR “warming”) AND “A* aegypti.” We included all articles, notes, early access papers and data papers written in English, and filtered the results according to document type. Our inclusion criteria were studies that (i) assessed how temperature or climate change affects *Ae. aegypti* ecology at any life stage (e.g., thermal preference, vectorial capacity); (ii) included *Ae. aegypti* in research on multiple species; and (iii) evaluated *Ae. aegypti* distribution. Thus, fieldwork, laboratory, and modeling studies were considered in our search. We excluded studies that (i) did not evaluate the effects of temperature or climate change per se; (ii) did not show statistical effects of temperature or climate change on *Ae. aegypti* populations; (iii) where the abstract content was sufficient but the full text was not available; and (vii) showed only the effect of CO_2_ concentration on *Ae. aegypti*. For the preferred reporting items for systematic reviews and meta-analyses (PRISMA) flow diagram showing the process steps and the filtering criteria [30], see Additional file 1: Figure S1.

### Data analysis

To analyze global collaboration networks and keyword networks, we conducted a bibliometric analysis using VOSviewer version 1.6.15 [31]. A collaboration network of countries was generated using the affiliation addresses of all the authors of a publication. To extract information on the number of publications by country, we used the country in which the institution of the first author was located for the bibliometrix R package. We merged England, North Ireland, Scotland and Wales into the UK to converge the names of countries used in the two bibliometric analysis programs (VOSviewer and bibliometrix R package). The node size illustrates the number of publications by country.

A keywords network was created using author keywords cited at least two times, and excluded the obligatory term “*Ae. aegypti*” and similar terms (e.g., “*Aedes-aegypti*,” “*Aedes aegypti*”). We chose to keep the terms temperature and climate change in the network. Although these terms were search terms, we sought to understand their connection to the other keywords. In this network, similar terms were merged (Additional file 1: SM2), which allowed a better view of the main topics covered by the documents. Node size in this network represents the number of occurrences of a keyword. To normalize the strength of the links between items in the cluster in both networks (global collaboration and keywords), we used the association strength method [31].

The bibliometrix R package [32] and biblioshiny interface were used to analyze the annual growth rate and the frequency of use of keywords over time. The annual growth rate was estimated by geometric progression, calculated through the compound annual growth rate (CAGR), which is given by:

$$CAGR={(\frac{Vfinal}{Vinitial})}^\frac{1}{t}-1$$

where,* v* is the number of documents in the initial and final year assessed and* t* is time. The rate is a geometric progression ratio that provides a constant rate over time for a given period (see the biblioshiny R package). Publication year was obtained by using bibliometrix and the graph was constructed using the ggplot2 package [33].

The most recent data published by global organizations were used to determine the numbers of dengue cases and the socioeconomic indices of each country. Dengue data were gathered from the World Health Organization (2017); HDI (2019) data were from the Human Development Report [21] and the Gini coefficient for 2010–2018 (the most recent available data during this period). GDP per capita for 2019 were extracted from the World Bank. To evaluate the correlation among the number of publications by country and dengue cases and socioeconomic indices (HDI, Gini coefficient and GDP per capita), we used the first author country in a Pearson correlation analysis. Where information on dengue cases or socioeconomic indices was incomplete for a country, they were not included in the correlation analysis. The analyses were carried out in the R environment [34].

## Results

### Annual increase in the number of publications

**Fig. 1:**
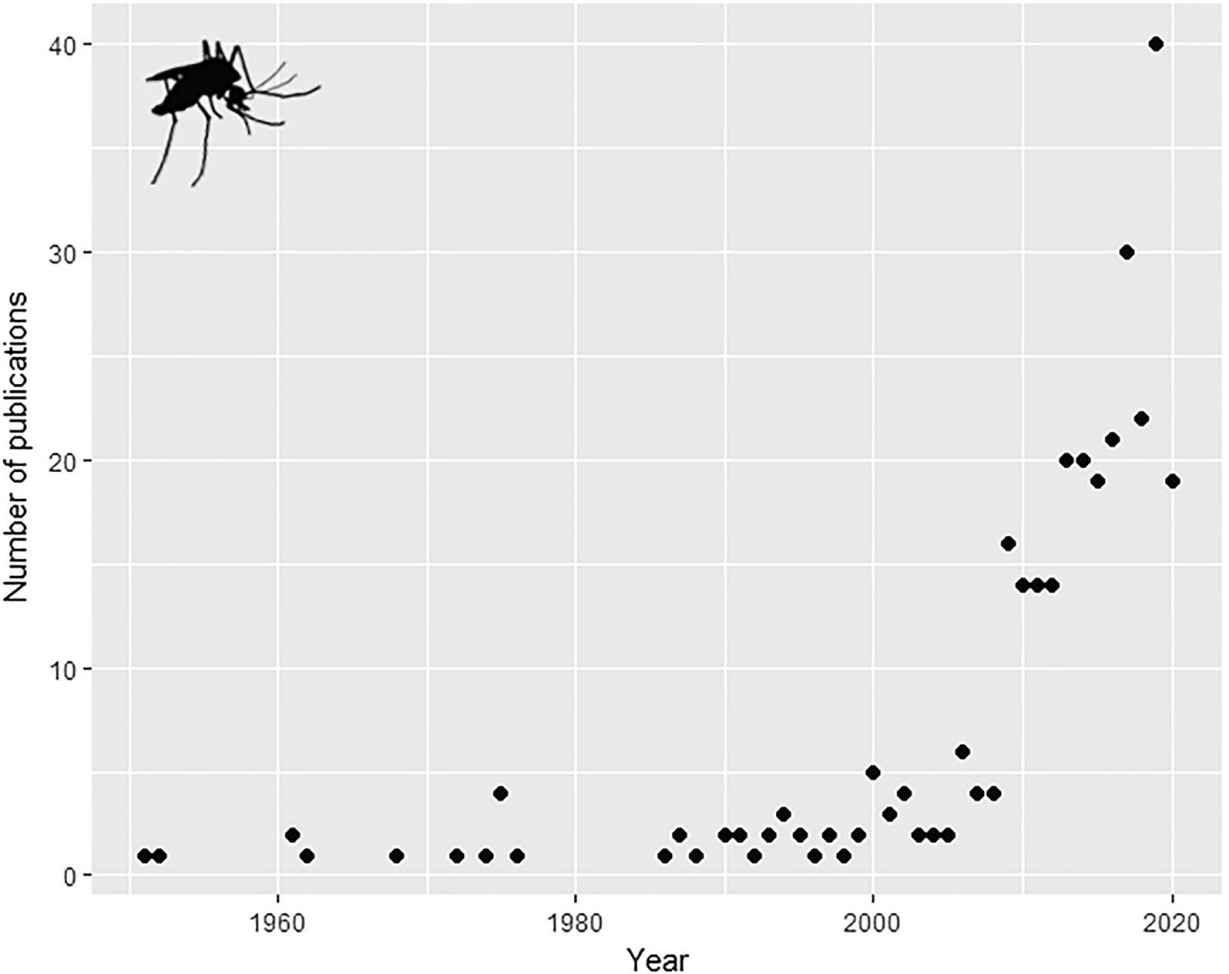
Number of studies published annually from 1951 to 2020 that assessed the effects of temperature, climate change or global warming on *Aedes aegypti*

We reviewed 317 studies that met our profile criteria. There was an observable increase in the number of studies during the period 1951–2020, and in particular after 2000; the majority of papers (*n* = 249) were published in the last 11 years of this period (Fig. 1). The average number of publications between 1951 and 1999 was 1.59 articles/year, but the average more than doubled during the following period (2000–2020). From 2000 to 2008, 3.56 articles/year were published, and there was a six-fold increase in publications from 2009 (average of 20.75 articles/year from 2009 to 2020). The year with the highest number of publications was 2019 (*n* = 40), although we assessed studies until October 2020. It is important to note that the number of publications in 2020 may have been affected by delays in publishing due to the impact of the COVID-19 pandemic. The annual rate of increase in the number of published studies for the period 1951–2020 was 7.26%.

### Keywords network and trends in keyword use over time

**Fig. 2:**
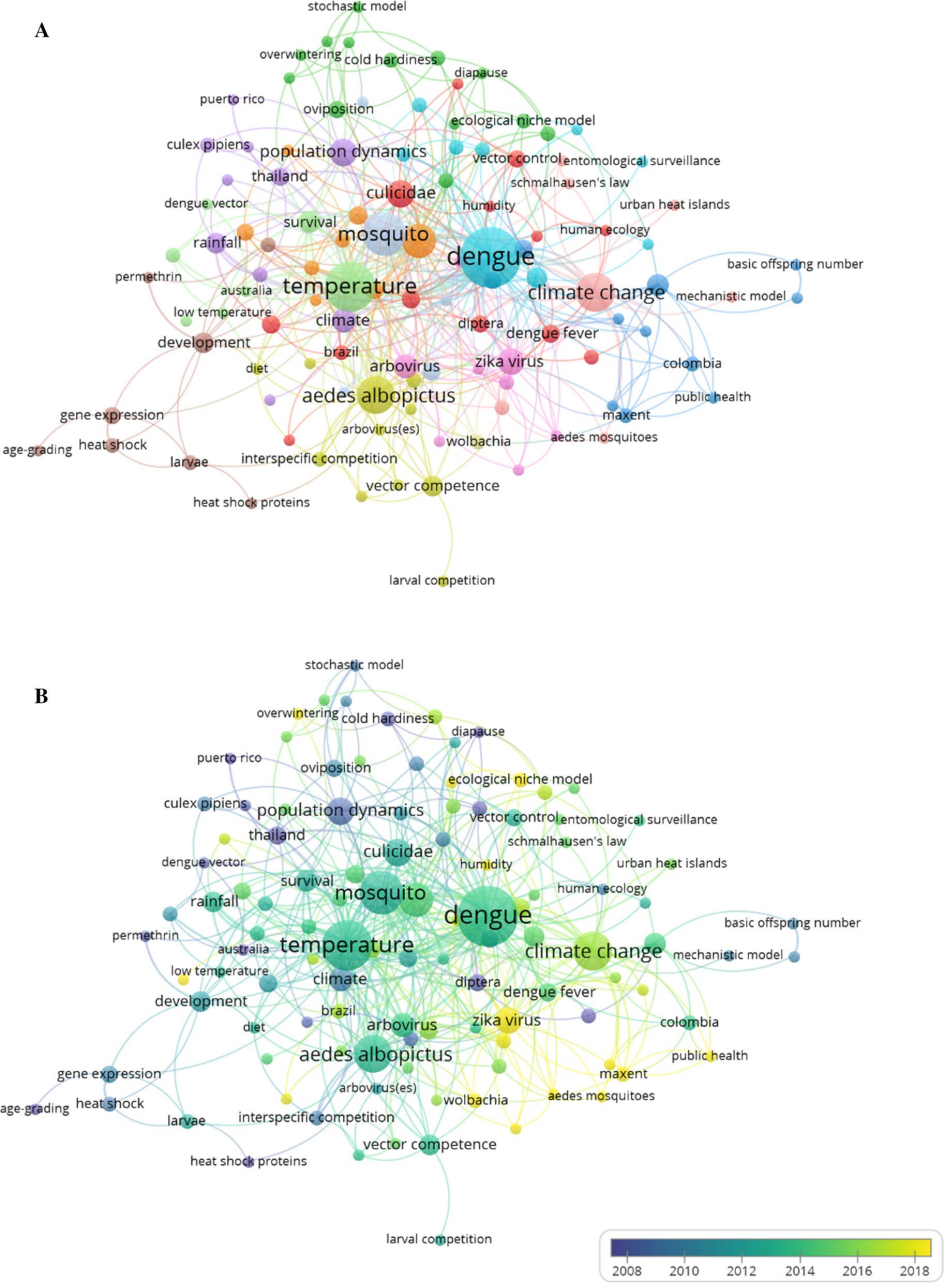
Keywords network **a** with cluster colors, and **b** over time. We considered keywords cited at least twice and excluded the obligatory term “*Aedes aegypti*” and similar terms. The node size represents the number of occurrences of a keyword and the links indicate the connections between keywords. N.B. The chikungunya node is located immediately below the Zika virus node

The keywords network consisted of 111 items and 13 clusters (Fig. 2a). Four terms were prominent in the center of the keywords network: dengue, temperature, mosquito and climate change. An analysis of associations (links) among terms showed that those related to disease, such as “infection” and “transmission,” belonged to the same cluster as dengue (total links, 53). The term temperature had 46 links and was connected with keywords related to species characteristics, such as “survival” and “development time,” as well as “mosquito,” which was connected to “larval habitats” and “abundance” (mosquito total links, 30). In contrast, the term climate change had more links (total links, 25) with terms that referred to mathematical models and statistical analysis (e.g., mechanistic model, kurtosis), and with “vectorial capacity” (Fig. 2a). The cluster formed by “*Aedes albopictus*” included terms related to “competition,” “arbovirus” and “vector competence.” The only term related to human health was “public health,” and despite the fact that it was not in the same cluster, it was linked with the term climate change.

**Fig. 3:**
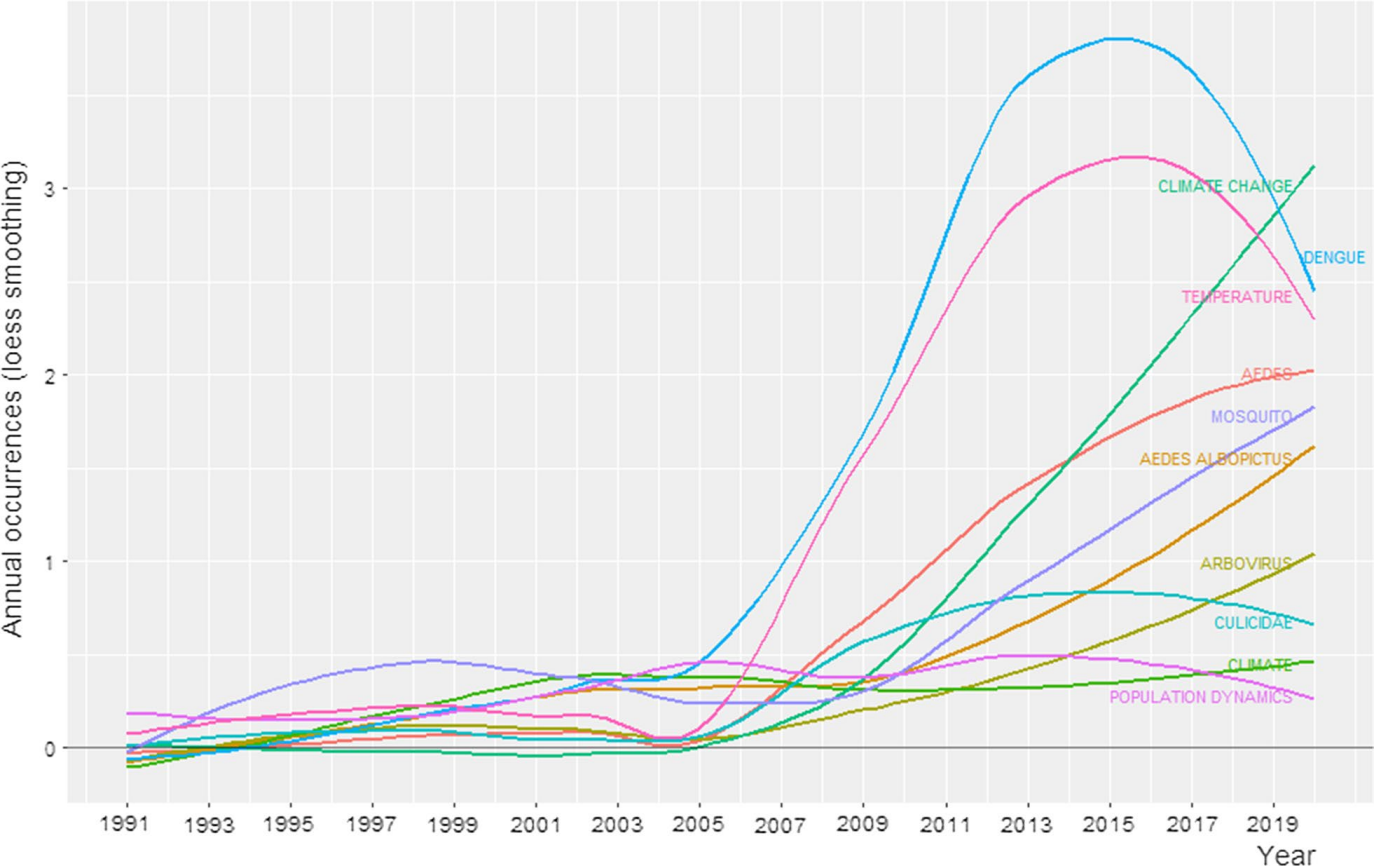
Changes in the frequency of use of the 10 most used keywords in the period from 1991 to 2020

With regard to the main pathogens transmitted by *Ae. aegypti*, dengue received a lot of scientific attention in 2013, while research that focused on chikungunya and Zika was mainly published in 2017 and 2018, respectively (Fig. 2b). Two of the topics of interest in our research, temperature and climate change, were also the focus of research in different years. Studies on temperature were mainly published in 2013, while climate change was a research topic in 2016 and 2017 (Fig. 2b). We found some remarkable changes in the frequency of the 10 most cited keywords over a period of almost 30 years (1991–2020). The number of publications including the term climate change increased with time, especially in the last 11 years (Fig. 3). Many publications included the terms dengue and temperature in this period (Fig. 2b), but fewer did after 2016. Other keywords such as mosquito, *Aedes albopictus* and arbovirus have also received a lot of attention more recently (Fig. 3).

### Global scientific publications and international collaborations

**Fig. 4:**
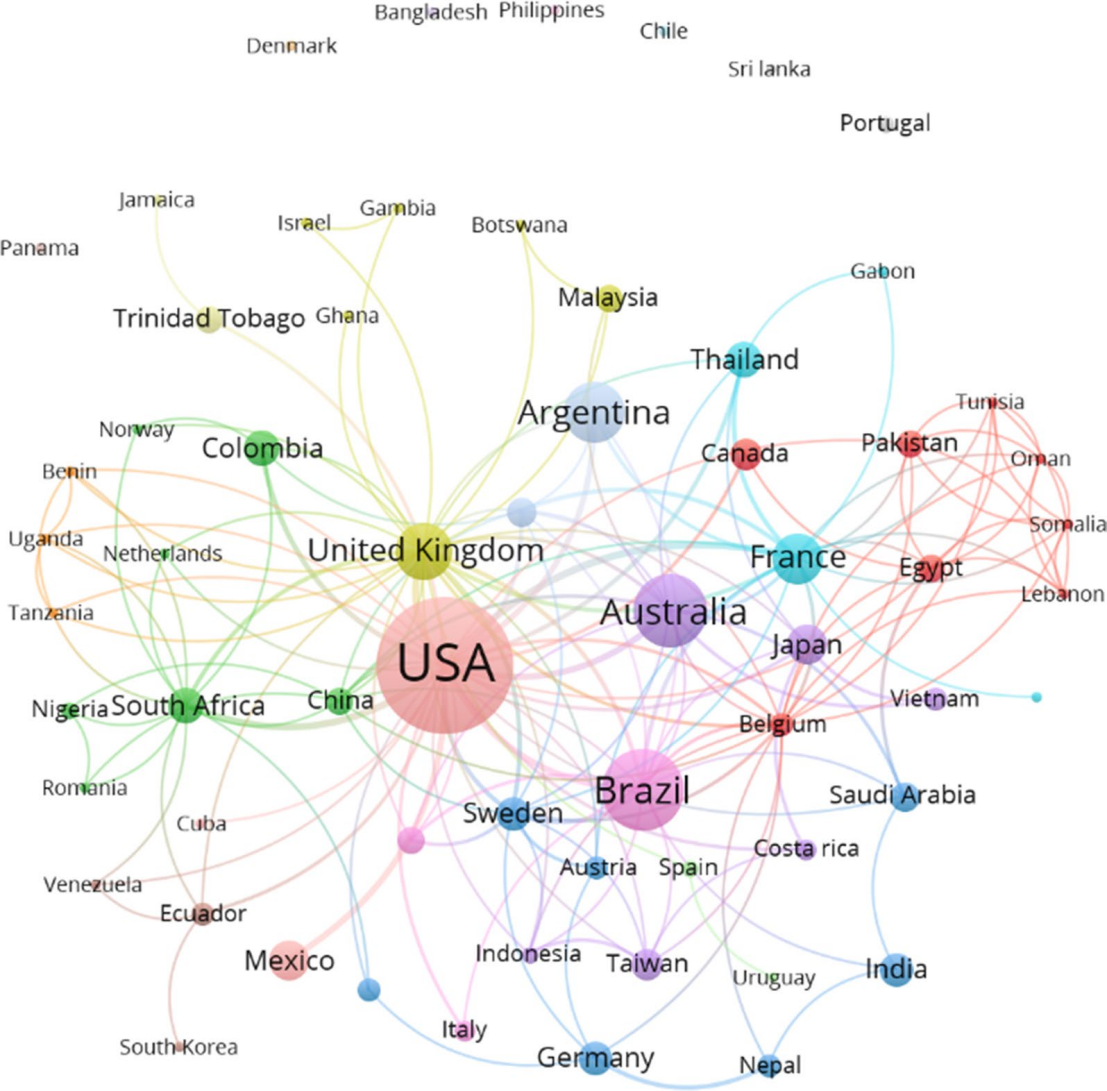
Collaboration networks between 63 countries with respect to studies on the effects of temperature or climate change on *Aedes aegypti*. Node size represents the number of publications per country and the links indicate co-authorship between countries

Sixty-three countries published and collaborated on the study topics. The countries that dominated the publications with respect to the affiliation of the first author were the USA (*n* = 91 papers), Brazil (*n* = 38 papers), Australia (*n* = 32 papers), Argentina (*n* = 21 papers), and the UK (*n* = 13 papers) (Fig. 4). These publications accounted for 61.5% of the total in this area of research. The publications from the USA accounted for ~ 30% of the total.

A collaboration network showing partnerships between the affiliation countries of all the authors comprised 20 clusters. Seven countries—Bangladesh, Chile, Denmark, Panama, Philippines, Portugal, and Sri Lanka—were not part of a cluster, which indicates that any collaborations were domestic. These countries had only one publication each in the period examined, with the exception of Portugal, which had two.

Countries with the largest number of publications did not necessarily have the largest collaboration network, with the exception of the USA and the UK. The USA had the most international collaborations, with articles published in collaboration with 32 other countries; in the case of the UK, collaborations were with a total of 29 countries. However, although some countries had fewer publications, their collaboration was high. For example, France published research undertaken in partnership with 19 countries, followed by Belgium (*n = *18 international collaborations), South Africa (*n = *14), Sweden (*n = *13), China (*n = *11), Egypt and Switzerland (both *n = *10). Brazil and Australia, which had high numbers of publications, had partnerships with 16 and 13 countries, respectively, while Argentina had international collaborations with five countries.

Of the five countries with the most publications, the UK was the only one for which the majority of publications (77%) were the result of collaborations with multiple countries. A similar result was found for some of the countries with fewer publications, such as France, from which there were six publications, five of which involved international collaboration. Other examples of international collaboration were given by Sweden (*n = *4) and Nepal (*n = *3), for which none of their publications were the result of domestic collaboration only. On the other hand, most publications from the USA, Brazil and Australia were authored by researchers based in those countries (87% for Brazil, 71% for USA, and 69% for Australia).

The correlations between the number of publications of a country, dengue cases (*r* = 0.077; *P* = 0.692) and socioeconomic index (HDI, *r* = 0.1765; *P* = 0.356; Gini coefficient, *r* = 0.2666; *P* = 0.162; GDP per capita, *r* = 0.3011; *P* = 0.112) were not significant.

## Discussion

The number of publications in English on the effects of climate change on *Ae. aegypti* increased significantly over the last decade and surpassed the number of published studies on the effects of temperature on this species. Five countries dominated this field of research (61.5% of the publications): three developed countries (the USA, UK and Australia) and two developing South American countries (Brazil and Argentina). With the exception of the UK, all of these countries have established *Ae. aegypti* populations. Taken together, our results show the need to intensify international research collaborations, especially between countries in the Global South. We discuss some mechanisms that have been effective in increasing international collaborations and can serve as examples for this.

### Publications and keywords trends over time

Following a general pattern of an exponential increase in the number of studies on climate change in recent years (1991–2014) [35], we also observed an increase in the number of publications from 2006 to 2020 that used the term climate change as a keyword. Climate change came into the worldwide spotlight as a topic in the late 1980s [36], and in 1990 the Intergovernmental Panel on Climate Change (IPCC) released its First Assessment Report (AR), which considered the risks of climate change for human health, including an increase in diseases and greater exposure to disease vectors. In 2011, Vasileiadou et al. [37] examined the impact of the then current IPCC-ARs on research on the effects of climate change on vector species. In our examination of the trend in the annual number of publications, we observed that the number was high in 2009; this may have been the result of the publication of IPCC-AR4 in 2007.

In the keywords network, although the terms temperature and climate change are prominent, the most frequently cited term was dengue. However, since some authors use the term “dengue vector” to refer to *Ae. aegypti*, we could not be certain that the research had focused on the dengue virus. In contrast to the term dengue vector, the frequencies in the keywords network of the names of other viruses transmitted by *Ae. aegypti*, specifically Zika and chikungunya, were lower. Commonly, research on temperature or climate change effects on these viruses investigates the vectorial capacity of *Ae. aegypti* [12, 38], or the extrinsic incubation period of the virus [14, 39]. However, the names of the viruses transmitted by this species are some of the few terms in the keywords network which are related to human health. Most terms in the keywords network are associated with vector biology, such as development rates, oviposition, and population dynamics.

Terms related to modeling studies had greater prominence in more recent publications (from 2014 onwards), and most were associated with the term climate change. Modeling studies have used climate change scenarios developed by the IPCC to investigate the range expansion of *Ae. aegypti* in specific countries, continents [40, 41], or worldwide [16, 42]. The results of these studies are extremely important for decision makers, as they can be used to develop measures to control species’ range expansions under climate change scenarios. Unfortunately, due to a lack of commitment of the governments of some key countries to global climate agreements [43], and the potential consequences of possible climate tipping points [44], the range expansion of *Ae. aegypti* is a likely scenario.

### Asymmetries in the number of publications of the five top-ranking countries

With respect to global scientific research on climate change, in a review undertaken in 2016 [35], the countries with the highest number of publications were the USA, UK, Germany, Canada, China, and Australia. However, in the present review, which is focused on *Ae. aegypti*, we found a different pattern, with two South American countries (Brazil and Argentina) among the top five (i.e., together with the USA, UK, and Australia). The large number of publications from some developing countries shows that, despite lower government investment in science and technology, their scientific contribution is high and fast growing [45, 46]; this reinforces the idea that the global balance of research is changing [25]. Of the three developed countries, there are three factors that may explain their research productivity in this field. Firstly, two of them, i.e., the USA and Australia, are among the 10 countries with the highest occurrences of *Ae. aegypti* [47]. Secondly, they have the economic resources for the development of research; and thirdly, they have invested in universities and research centers with a focus in this field (e.g., the University of Florida and the Centers for Disease Control and Prevention in the USA; the University of Melbourne and James Cook University in Australia; the London School of Hygiene and Tropical Medicine in the UK).

A country’s population size may also have an effect on the number of publications if one follows the reasoning that more populated countries (human resources) may have more graduates and more researchers in a given field. We did not include population size as a predictor in our analyses, yet the five top-ranking countries do provide a good example of this relationship. These countries include three with respectively small to moderate human populations (Australia, 26 million people; the UK, 67 million people; Argentina, 45 million people), compared with the more populous countries (the USA and Brazil). In addition, countries with large human populations (e.g., Indonesia and Pakistan), and the most populous nations (China, India), had few publications. Finally, human population size alone does not seem to be a good proxy for the number of publications, given that scientific research requires human resources, infrastructure and funding [25, 48].

Among the developing countries with the most publications in this area of research, Brazil is among those that are most impacted by viruses transmitted by *Ae. aegypti*. According to the Pan America Health Organization, in 2019, the total number of dengue cases in the country was 2,248,570, almost 2 million more than in the previous year. Over a 20-year period (1995–2015), the number of cases of dengue reported in Brazil represented more than half of those reported in the Americas [49]. In Argentina, the number of dengue cases has increased markedly in recent years, from 557 cases registered in 2017 to 59,358 in 2020 [50]. Although there was no correlation between the number of dengue cases and the number of publications of a country, the high number of cases in these developing countries may be one of the reasons why they are among the most productive with respect to the number of publications in this field of research. In addition, they have consolidated research groups and large research centers with this theme as a focus (Brazil—Fiocruz; Universidade de São Paulo; Universidade Federal de Minas Gerais; Argentina—Universidad de Buenos Aires; Universidad Nacional de Córdoba).

### Falling behind

The research scenario is different for some other countries with a high number of inhabitants infected by the dengue virus. Some countries in Asia and South America had few publications, despite the fact that they have high numbers of cases of dengue. For example, India, Sri Lanka and Vietnam had more than 150,000 dengue cases registered in 2017 and Colombia 78,298 dengue cases in 2020. Other countries had no publications at all in this area of research, including Paraguay, Bolivia and Myanmar, which had, respectively, 223,082 (2020), 85,130 (2020) and 7729 (2017) recorded dengue cases. Some of these countries are located in regions that are currently considered the most suitable for the development of *Ae. aegypti*, such as Southeast Asia, South America, and West and Central Africa. Climate change scenarios also predict that these areas will see the greatest range expansions of *Ae. aegypti* [15].

International collaboration is essential to increase the visibility of countries which have few or no publications in this area of research [22]. The USA and UK had the highest number of international collaborations, and some of the other countries with a high number of research collaborations have English as their official language. English is the universal language of science [51], and researchers in English-speaking countries are more likely to collaborate internationally [23, 52]. Moreover, as reported here, most reviews on the publication of scientific research show a bias towards papers written in English (but see [53]), which results in an underestimation of publications that are mainly from developing countries. However, the issue of the language of use in the scientific world is being rethought. For example, the Journal of Applied Ecology encourages authors to write the abstract of their articles in their native language, or even provide the entire article in it (Additional file 1: Figure S1). In addition, that journal suggests that reviewers should not ask non-English-speaking authors to have their manuscript edited by a native speaker of English [54] as this increases their publication costs. Although the majority of international collaborations in this area are from the USA and UK, at least one country on each continent (i.e., Australia, China, UK, South Africa, USA) had a high number of international partnerships. These countries can serve as hubs for boosting research on their respective continents.

We found a distinct profile of collaborations between regions or continents. In South America, there was limited collaboration between Brazil and Argentina, and none of the countries of this content had collaborated with African countries. Colombia, Venezuela, and Ecuador had collaborations with South Africa, the country on the African continent with the most international links. Central American countries collaborated only with other countries in the region, or with the USA, so it is suggested that they should extend their collaboration to countries with similar climates, which may face similar problems in the face of the range expansion of *Ae. aegypti*.

Asian countries were not amongst those with the highest number of publications, despite their long experience in surveillance and dengue control, which for some, such as Taiwan, began as early as 1988 [55]. A recent review [47] lists Taiwan as having the highest number of records of *Ae. aegypti* occurrences in the world. This may reflect its long history of, and public investment in, mosquito surveillance as a measure of the extent of its population. Regardless of its low publication output compared to other countries, Taiwan had a number of international collaborations (*n = *7), and the same was seen for Indonesia (*n = *6), Thailand (*n = *6), and China (*n = *11). Southeast China is one of the most populous regions of that country and is projected to experience a range expansion of *Ae. aegypti* in climate change scenarios for 2050 [15], and the number of publications on this topic have increased since 2020. Overall, though, there have been only a few publications relating *Ae. aegypti* to climate change in China and other Asian countries. Although within-country surveillance and reporting may be adequate for disease management, boosting the number of academic publications promotes both greater global understanding of the nature of the problem and management strategies.

In publications from Europe, the occurrence of *Ae. albopictus* is more frequently the focus than that of *Ae. aegypti* [47, 56], notwithstanding the UK’s prominence with respect to publications on *Ae. aegypti* and climate change. However, information provided by European research is essential for climate change scenarios, as range expansion of *Ae. aegypti* in this region has been predicted, in particular for the Iberian Peninsula, Greece, and Italy [15]. Few studies on the effect of temperature or climate change on *Ae. aegypti* have been undertaken in these countries. We did not find any research records for Greece, and Portugal was one of the countries that only published work undertaken by scientists based in the country. Other countries that also only published research carried out without international collaboration included Sri Lanka, Bangladesh and the Philippines. The Representative Concentration Pathway 6.0 scenario, in which radiative forcing is stabilized after the year 2100, predicts an increase in the distribution of *Ae. aegypti* by 2050 [56]. Therefore, the situation in these countries deserves more attention.

### Suggestions

Some inclusive mechanisms can be used to boost international collaborations and increase the visibility of research from the Global South and poorer Asian and African countries. These mechanisms include (i) research networks, for example the Thematic Research Network on Data and Statistics, which is a United Nations initiative for sustainable development that seeks solutions to improve data usage to ensure sustainable development; and the Latin American Brain Mapping Network, which is a training and exchange program designed to promote improvement in neuroimaging and systems neuroscience in Latin America [57]; (ii) internationalization programs, whereby governmental agencies fund work exchange amongst researchers and boost international collaboration [e.g., PrInt, a program of the Coordenação de Aperfeiçoamento de Pessoal de Nível Superior (CAPES), a Brazilian governmental agency]; (iii) academic mobility programs, for the mobility of academics seeking partnerships and exchange of information and ideas with researchers from different countries [58]; (iv) increasing the number of editors representing the Global South on the editorial boards of journals [54]. Moreover, it is critical to enhance diversity, equity and inclusion—also in leadership—in research on climate change and vectors, and to reduce the distance between research and implementation at all levels and in all mechanisms involving collaboration.

In addition to research collaborations, it is important that countries commit to global climate agreements. Efforts to minimize greenhouse gas emissions, such as land restoration and combating deforestation, are also useful for reducing the occurrence of *Ae. aegypti* and other insect vectors [59, 60]. These actions can help to minimize the invasion of anthropophilic mosquitoes and thus decrease the incidence of infectious diseases [61, 62].

## Conclusions

Evaluating the effects of temperature or climate change on *Ae. aegypti* has attracted increasing attention from the scientific community in the last 30 years. At present, there is more research on the effects of climate change on *Ae. aegypti*, whereas the number of publications that assess temperature effects on this mosquito has been decreasing. Two South American countries are amongst the top five in terms of number of publications, and show a high level of international collaboration in this research area. They could act as hubs for other countries in the Global South, including poorer Asian and African countries, to boost research in these regions. Many countries in Southeast Asia, South America, and West and Central Africa, which are critical with respect to the occurrence of *Ae. aegypti*, have few publications in this area, so researchers should consider increasing their collaboration with these countries in the future. An increase in collaborations would help to provide the knowledge required for these countries to design management strategies in response to the predictions of climate change scenarios.

## Supplementary Information

**Additional file 1: Figure S1**. PRISMA flow diagram. **SM2.** Similar terms merged in bibliometric analysis methods using VOSviewer version 1.6.15.

## Data Availability

Not applicable.
