# Supplementary material for: Global trends in research on the effects of climate change on Aedes aegypti: international collaboration has increased, but some critical countries lag behind
Source: Parasit Vectors. 2022 Sep 29;15:346. doi: 10.1186/s13071-022-05473-7 (PMC9520940; doi:10.1186/s13071-022-05473-7)
Supplement: Supplementary file 1 — Additional file 1: Figure S1. PRISMA flow diagram. SM2. Similar terms merged in bibliometric analysis methods using VOSviewer version 1.6.15. [file 13071_2022_5473_MOESM1_ESM.docx]

**Supplementary material**

SM1. PRISMA flow diagram

Studies included in quantitative synthesis (meta-analysis)
(n = 317)

Studies included in qualitative synthesis
(n = 317)

Full-text articles excluded, with reasons
(n = 14)

)

Full-text articles assessed for eligibility
(n = 331)

Records excluded
(n = 827)

Records screened
(n = 1158)

Records after duplicates removed
(n = 1361)

Additional records identified through other sources
(n = 0)

## Identification

## Eligibility

## Included

## Screening

Records identified through database searching
(n = 1361)

SM2. Similar terms merged in bibliometric analysis methods using *VOSviewer* version 1.6.15.

**i) Keywords network**

- we used keywords that were cited at least 2 twice
- trivial keywords and similar were removed, as follow: ‘*ae. aegypti*’ a*edes-aegypti*’ and ‘*aedes aegypti*’
- a merge term was performed, using thesaurus. We kept the term that appears more times. The following terms have been replaced:

‘*ae. albopictus*’ replaced to ‘*Aedes albopictus*’

‘albopictus’ replaced to ‘*Aedes albopictus*’

‘dengue virus’ replaced to ‘dengue’

‘matemathical modelling’ replaced to ‘mathematical model’

‘mosquitoes’ replaced to ‘mosquito’

‘mosquito-borne diseases’ replaced to ‘mosquito-borne disease’

‘ovitrap’ replaced to ‘ovitraps’

‘zika’ replaced to ‘zika vírus’

The total keywords was 632, after application the filters 111 were used in keywords network.

- type of analysis: *co-occurrence*
- unit of analysis: *author keywords*
- counting method: *full counting*
